# Encapsulation of *Lacticaseibacillus rhamnosus* by Extrusion Method to Access the Viability in Saffron Milk Dessert and Under Simulated Gastrointestinal Conditions

**DOI:** 10.1002/fsn3.4510

**Published:** 2024-10-23

**Authors:** Mohammad Ganje, Seyed Saeed Sekhavatizadeh, Fatemeh Teymouri, Mostafa Gilkheiri, Bentalhoda Rahmani

**Affiliations:** ^1^ Department of Agriculture, Minab Higher Education Center University of Hormozgan Bandar Abbas Iran; ^2^ Department of Food Science and Technology Fars Agricultural and Natural Resources Research and Education Center, AREEO Shiraz Fars Iran; ^3^ Department of Food Science and Technology Neyshaboor Technical and Vocational University Neyshaboor Iran; ^4^ Department of Food Science and Technology Islamic Azad University Noor Iran; ^5^ Department of Food Science and Technology Kashmar Academic Center for Education, Culture and Research Kashmar Iran

**Keywords:** extrusion, *Lacticaseibacillus rhamnosus*, saffron milk dessert, simulated gastrointestinal condition

## Abstract

The effectiveness of probiotics in delivering health benefits may be associated with their capacity to maintain a minimum concentration of 10^6^ CFU/g during food storage and to successfully colonize the gastrointestinal tract (GI). *Lacticaseibacillus rhamnosus* (LR) is a probiotic that does not exhibit adequate stability under harsh conditions. To enhance the survival capacity of LR during gastrointestinal storage, alginate (ALG) was used as a primary encapsulating layer through extrusion microencapsulation. Subsequently, camelina seed mucilage (CSM) and camelina seed protein (CSP) were applied as secondary layers at varying concentrations (0%–4%). Among the tested formulations, ALG‐CSM‐CSP (1.5%, 4%, 4% w/w) exhibited significantly higher encapsulation efficiency (94.15%) and provided appropriate LR encapsulation in SEM image. Three saffron milk desserts (SMD) containing free LR (FLR), microencapsulated LR (MLR), and a control (C) were prepared, followed by physicochemical and microbiological assessments of the samples. The result showed that at the end of storage, SMD had the lowest pH (6.21), the highest acidity (30°D), and maintained the permissible limit of probiotic bacteria (6.7 log cfu/mL) among the samples. In GI, the MLR and FLR survival rates were 43% and 45.4%, respectively on the 14th day of storage, respectively. The MLR hardness (313.70 g), adhesiveness (2.01 mJ), chewiness (9.36) and gumminess (58.8) had the greatest values among the samples. Moreover, SEM images showed a relatively denser structure for MLR. In conclusion, this study highlights the potential of CSM and CSP to protect probiotics, offering valuable insights for developing new functional foods with improved survival during storage and GI.

## Graphical abstract

Camelina mucilage and protein has great potential as edible hydrocolloids for microencapsulation of active agents (such as probiotic bacteria). The microencapsulation of *Lacticaseibacillus rhamnosus* has enhanced its resistance to heat, salt, and acidity. Microencapsulated *Lacticaseibacillus rhamnosus* in milk saffron desert demonstrates better survivability during storage time and in simulated gastrointestinal condition.

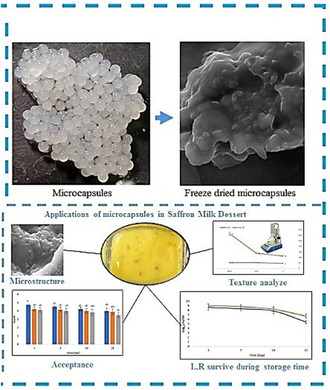

## 1 Introduction

Probiotics are beneficial microorganisms that provide benefits when ingested in significant amounts (10^6^ CFU/g) and promote consumer health. Currently, *Lactobacillus, Leuconostoc*, and *Streptococcus* belong to the group of microbial probiotics in the lactic acid bacteria genera (Vera‐Santander et al. 2023).

*Lacticaseibacillus rhamnosus* (LR) belongs to the lactic‐producing, Gram‐positive, and nonspore‐forming bacteria. It has been classified as a beneficial probiotic for the gut (Fuad et al. 2023). Moreover, it is used as a probiotic in puddings (Helland, Wicklund, and Narvhus 2004), dairy desserts (Dokoohaki, Sekhavatizadeh, and Hosseinzadeh 2019), yogurt desserts (Hashemi, Gholamhosseinpour, and Abedi 2021), drinkable desserts (Taheri et al. 2022), and pumpkin frozen desserts (Szydłowska and Kołożyn‐Krajewska 2019). Although the survival of probiotic bacteria is not expected under harsh conditions, the use of encapsulation techniques is recommended to enhance their stability. Probiotics must maintain viability during food storage, transport, gastric digestion, and provide beneficial effects on the GI tract (Ni et al. 2023). Probiotics have been encapsulated using various materials or biopolymers. One of the materials is ALG. ALG suffers several disadvantages because of its lack of mechanical and chemical stability. Ionic and covalent cross‐linking between alginate and coating materials functions as a secondary wall material for cell encapsulation, representing a strategy to address the associated disadvantages (Simó et al. 2017). The optimal capsule size and quality were achieved with a sodium alginate concentration of 1%, yielding capsules with rounded shapes, uniform sizes, and soft yet resistant structures (Tashybayeva et al. 2024). Proteins and polysaccharides can be potential candidates for the second layer wall materials. Typically, mucilages are comprised of polysaccharide hydrocolloids with distinctive structural and physicochemical properties (Waghmare, Moses, and Anandharamakrishnan 2022). The structural properties of polysaccharides are intricately linked to their raw material sources and the methods used for their preparation, which in turn affects their biological activities. For instance, polysaccharides derived from *Lactarius deliciosus* have been shown to enhance the levels of intestinal *lactobacilli* (Dong et al. 2024). Moreover, carbohydrates can enhance gastrointestinal health. For instance, rice resistant starch derived from natural rice that contains a high concentration of resistant starch can lead to an increase in the abundance of beneficial probiotics (such as *Lactobacillus* and *Ruminococcus*) and a decrease in the population of harmful bacteria (including *Desulfovibrio* and *Prevotella*; Ren et al. 2024).

Incorporating mucilage in food systems enhances the stability of target compounds and offers advantages in delivery properties. Mucilage is considered suitable for nano‐ and microencapsulation of bioactive compounds and bacteria. The molecular weight of the coating material has a notable impact on the oxidative stability of the core due to diffusion‐related effects. Mucilages exhibit high tolerance to a broad spectrum of temperatures and pH, making them excellent alternative biomaterials for encapsulation processes (Waghmare, Moses, and Anandharamakrishnan 2022). The combination of protein and mucilage may offer a viable solution. Proteins that facilitate strong interactions between carboxyl (COO^−^) and amino (NH_3_^+^) groups can lead to the formation of stable complexes (Kavousi, Fathi, and Goli 2018).

One of the oilseed plants is Camelina (*Camelina sativa*), which belongs to the *Brassicaceae* family (Mondor and Hernández‐Álvarez 2022). Camelina mucilage primarily comprises polysaccharides (∼80% w/w), along with proteins (10%–15% w/w) and minerals (∼6% w/w). The polysaccharides consist of xylose, rhamnose, glucose, galactose, and arabinose, with the acidic pectin‐like portion predominantly in the form of gal‐hamno‐galacturonan (Fabre et al. 2020).

Saffron milk dessert (SMD) is a famous culinary delight. Saffron (*Crocus sativus*) is a traditional herb primarily cultivated in Spain, Iran, Greece, India, and Italy. It is highly valued not only for its aromatic and coloring properties but also for its bioactive compounds that offer various health benefits. Saffron is obtained by harvesting the flowers and extracting metabolites from the dried stigmas (Rahaman et al. 2023).

We hypothesized that the microencapsulation could improve LR viability under harsh conditions and during storage time. To the best of our knowledge, the production and incorporation of MLR using camelina mucilage and protein into SMD have not been explored. Therefore, in the present study, hydrogel beads were produced using the extrusion method, with alginate serving as the first layer and camelina protein and mucilage at varying concentrations forming the second wall materials. This composition was employed for the encapsulation of LR. To identify the optimal formulation for the second layer of beads using CSM and CSP, various concentrations of CSM and CSP were tested as wall materials. The morphology, microencapsulation efficiency, resistance to heat stress, and survival under acidic and salt conditions were evaluated to determine the best formulation of bead samples. Subsequently, the ideal bead was incorporated into a dairy dessert, and an assessment was conducted on the physicochemical properties and viability of the beads during storage and gastrointestinal transit.

## 2 Materials and Methods

### 2.1 Materials

Freeze‐dried *Lacticaseibacillus rhamnosus* ATCC 53103 was obtained from the Persian Type Culture Collection, Tehran, Iran. MRS agar, sodium citrate, MRS broth, pepsin, and peptone water were procured from Merck (Merck, Darmstadt, Germany). Sodium alginate (CAS No 9005‐38‐3) with medium viscosity was purchased from Sigma Company (Sigma, Steinheim, Germany). Camelina seeds were obtained from Saharkhiz, a local market in Shiraz, Iran. Analytical‐grade chemicals were supplied by Merck (Darmstadt, Germany).

### 2.2 Extraction of Camelina Seed Mucilage

For the extraction of camelina seed mucilage, Ubeyitogullari and Ciftci (2020) method was used. Briefly, camelina seeds were immersed in deionized water (Dadwal & Gupta) at a water‐to‐seed ratio of 15:1 and agitated in a closed glass vessel at 85°C for 3 h with a mixing rate of 600 rpm. Subsequently, the seeds were separated by centrifugation and filtered through Whatman filter paper no. 1. Initially, the mixture was subjected to vacuum filtration with a cloth. Then the seeds were rinsed twice with hot deionized water (85°C). For the final separation, the filtrate was centrifuged at 4000 *g* for 10 min. The supernatant was dried overnight in an oven set at 100°C. The resulting dried mucilage was milled using a Moulinex miller (France) to obtain mucilage powder. The mucilage powder was kept at ambient temperature, until it was used for gelation (Ubeyitogullari and Ciftci 2020).

### 2.3 Preparation of Protein Isolate

Camelina seed was defatted using hexane mixed with a ratio of 1:5 (w/v) and left for 5 min. The sample was filtrated with Whatman filter no. 1 paper. Before protein isolation, samples were air‐dried and maintained at −20°C. The pH solubilization was used for protein extraction from defatted samples. Protein isolates were obtained from defatted camelina seed meals based on the method of Ngo and Shahidi (2021). The defatted samples were added to DW (3.0% w/v). The mixture was agitated with a shaker for 30 min. Then, the pH was adjusted to 12.0 by adding NaOH 2 M and agitated for another 60 min at 27°C. Under the same conditions, the residues were re‐extracted twice with distilled water. The supernatants were combined, and the pH was decreased to 4.5 by the addition of HCl 2 M. The protein was precipitated by centrifuging at 10,000 *g* for 30 min at 4°C. The protein sediment was gathered and then washed twice with DW. The separated protein was redissolved in DW, and the pH was adjusted to 7.0 by adding sodium hydroxide 1 M. The extracted proteins were freeze‐dried and stored at −20°C for further analyses (Ngo and Shahidi 2021).

The protein that was extracted was subjected to freeze‐drying and was subsequently stored at a temperature of −20°C for additional assessment.

### 2.4 Bacterial Preparation

LR cultivation under anaerobic conditions at 30°C for 48 h was performed using the novel selective medium modified rhamnose 2,3,5‐triphenyl tetrazolium chloride–LBS–vancomycin (M‐RTLV). The culture media was centrifuged at 2264 *g*, at 4°C for 10 min. The culture media was washed twice with sterile saline before being re‐suspended in 0.1% w/v peptone water.

### 2.5 LR Encapsulation Procedures

Following activation of LR, the culture was subjected to centrifugation at 2800 *g* for 10 min. The LR final volume was 5 mL, containing an approximate bacterial count of 9.93 × 10^9^ CFU/mL in the MRS broth. The application of microencapsulation was accomplished by employing the extrusion method. In this process, the LR culture (5 mL) was combined with a 1.5% w/v sodium alginate solution (15 mL) and then injected into a sterile 0.1 M CaCl_2_ solution through a 0.11 mm needle. After 12 h of cooling, the beads were washed with 0.1% w/v peptone water, followed by gentle stirring at 1 g for 40 min in CSM and CSP solutions (Table S1), separately. Finally, after several washes, the beads were rinsed with sterilized 0.1% w/v peptone water (Dokoohaki, Sekhavatizadeh, and Hosseinzadeh 2019).

### 2.6 Encapsulation Efficiency

The encapsulation yield (EY) was calculated and is demonstrated in Equation (1):

(1) $$\mathrm{EY}=\frac{\mathrm{Log}\;N}{\mathrm{Log}\;N_{0}}\times 100$$

In this formula, “*N*” represents the number of LR obtained after encapsulation (CFU/g microspheres), and “*N*_0_” is the number of LR initially applied to prepare the beads (CFU/g exists in mixed alginate).

### 2.7 Color

The values of bead and dessert color were determined using a Konica Minolta CM‐5, Hunter Lab (Osaka, Japan). The colors were measured in terms of (L*) for lightness/darkness (0 for black and 100 for white), (b*) for blueness (−b for blueness, +b for yellowness), and (a*) for greenness (−a for greenness, +a for redness).

### 2.8 Heat Resistance of MLR and FLR

The thermal durability of FLR and MLR (approximately 8–9 log CFU/mL) was examined at 72°C for 0, 3, 6, 9, 12, and 15 min. Following heat stress, LR survival was determined by serial dilution and cultivation on M‐RTLV agar. LR viability was assessed using serial dilution and culture methods.

### 2.9 Survival in Salt and Acid Conditions

A 15% NaCl w/v solution with glycine‐HCl buffer (pH 1.5) was used to assess the survival of MLR and FLR. Samples were collected at 0, 30, 60, and 90 min and then serially diluted. The pour plate method was employed to culture LR, with plates incubated at 37°C for 2 days.

### 2.10 Microscopy and SEM

The aspect ratio, shape, and size were examined using a digital microscope (Olympus BX51, Japan). These images were analyzed with Micro‐Measure software (ver 1.07). The aspect ratio was calculated using Equation (2):

(2) $$\text{Aspect Ratio}=\frac{\text{Major Axis}\;}{\text{Minor Axis}\;}$$

After determining the optimal bead wall formulation, SEM analysis was carried out in the selected bead. Moreover, the cross‐sections of dessert samples on the 1st and 21st days of storage were coated with gold to enhance conductivity and mechanical stability under vacuum. They were then analyzed using SEM (JSM‐5800LV, JEOL, Japan) to observe the outer layer structure of the beads.

### 2.11 Preparation of Probiotic SMD

Skim milk powder was dissolved in NaCl 1 M to achieve a final concentration of 10% w/w. The mixture was stirred at 300 rpm using an agitator. Sucrose and modified starch were added to the milk, and the mixture was stirred for 30 min at 27°C. A solution of k‐carrageenan in NaCl 0.1 M was prepared and agitated for 30 min at 27°C before pasteurization at 75°C for 10 min. In the final formulation, the dessert contained 8 g of sucrose, 0.1 g of k‐carrageenan, saffron powder, and 2 g of modified starch per 100 g of the product. The samples were then blended for an additional 5 min at 50°C (Szwajgier and Gustaw 2015). Next, three equal parts of the dessert were applied in this research. The initial concentration of LR in FLR and MLR desserts before hot filling was approximately 8.82 Log CFU/g. The other dessert served as a control (C). All samples were packed at 80°C, immediately cooled with cold water for 1 min, and stored at 4°C. Microbiological, sensory, and physicochemical properties, including texture parameters, color, titratable acidity, pH, and LR survival, were assessed during storage (Dokoohaki, Sekhavatizadeh, and Hosseinzadeh 2019).

### 2.12 Titratable Acidity and pH

Titratable acidity in SMD samples was determined by acid–base titration, and the result was reported as a Durnic degree. The pH of the supplemented and control SMD was measured using a digital pH meter (GPRT‐1400‐AN; Greisinger Electronic GmbH, Germany).

### 2.13 LR Survival Ability During GI Condition

For this purpose, 1 g of MLR and an equivalent amount of FLR‐SMD were separately placed into sterile tubes containing 9 mL of saline (0.5% w/v). HCl 1 N was used to standardize the pH of all samples to a range of 1.4–1.9, suitable for pepsin (porcine, gastric mucosa, Sigma) and lipase (Fluka 62305). Lipase and pepsin were added at concentrations of 0.9 and 300 mg/L, respectively. The samples were placed in a shaking incubator (Lab Tech, Korea) at 3 g, 37°C for 2 h (gastric phase). To adjust the pH of the sample to a range of 4.3–5.2, an alkaline solution consisting of 14 g of Na_2_HPo_4_ in 150 mL of NaOH 1 N was used. Pancreatin 1750 (pancreatin pancreatic, Sigma) and Bile (bovine bile, Sigma B838) were prepared at concentrations of 1 and 10 g/L, respectively. Subsequently, an alkaline solution was employed to elevate the pH levels of the samples to a range of 6.5–7.5. The same concentrations of bile and pancreatin were added to the supplemented SMD. They were placed inside a shaker incubator (3 g) at 37°C for 2 h (second intestinal stage). Culture and counting of LR were performed on MLR and FLR‐SMD at 0.5, 1, 2, 4, and 6 h (Pourakbar et al. 2023).

### 2.14 Storage Stability on SMD

During storage, the survivability of FLR and MLR was assessed by supplementing MRS with 4 μg/mL erythromycin (Sigma). To achieve this, 1 g of MLR was combined with 9 mL of sterile MRS broth, and 0.1 g of FLR was combined with 9.9 mL of sterile MRS broth. Cultivation of FLR was carried out for 28 days, with samples taken every seven days during storage at 4°C in the mentioned culture media.

### 2.15 Texture

The Brookfield texture analyzer (CT3 4500, USA) was used to assess the texture profile analysis (TPA) of the dessert samples on the initial and final days after production. The cylindrical probe (TA11/1000) was used for this assessment. The diameter was 20 mm, the height of the sample was 30 mm. The penetration depth was 20 mm based on the following speeds: pre‐test 1 mm/s, test 1 mm/s, and post‐test 10 mm/s. The textural parameters, including hardness (g), chewiness (mJ), cohesiveness, gumminess (g), and adhesiveness (mJ) were obtained from the texture analyzer output. TPA was conducted for every sample at 25 ± 3°C (Karimi, Sekhavatizadeh, and Hosseinzadeh 2021).

### 2.16 Survival Rate Calculation

For the calculation of the survival rate, Equation (3) was used.

(3) $$\text{survival rate}\;\left(\%\right)=\frac{\mathrm{CFU}\;\mathrm{Log}\;N}{\mathrm{CFU}\;\mathrm{Log}\;N_{0}}\times 100$$

After the harsh treatment or during the storage time, the number of live probiotics in CFU/mL was indicated by the value of *N*, while *N*_0_ indicated the count of viable probiotics in CFU/mL before harsh treatment or at the beginning of the experiment.

### 2.17 Statistical Analysis

Statistical analysis was conducted using SPSS software (version 21.0). The results were presented as means ± SD. For statistical analysis, a one‐way analysis of variance followed by Duncan's multiple range test was employed with a 95% confidence level to detect significant differences among the samples' means.

## 3 Results and Discussion

### 3.1 Morphology, Size, and Encapsulation Efficiency of Beads

In this work, the obtained beads diameters were 3299.90 ± 54.26–3349.30 ± 116.48 (μm; Table 1). Dadwal and Gupta (2023) showed that the beads diameter by the extrusion method exceeded 2 mm (Dadwal and Gupta 2023). The average aspect ratios were from 1.12 to 1.18. Various factors may influence the hydrogel beads shape and diameter. These factors include type, extruder nozzle diameter, viscosity, CaCl_2_ concentration, and distance between syringe and solution (Ni et al. 2023). The beads volume increased and their diameter expanded because of water absorption and swelling of CSM and CSP. Moreover, the bead wall served as an intact physical barrier.

**TABLE 1:** MLR layer dimension, color parameters and encapsulation efficiency.

Samples	Layers dimension (μm)		Color parameters			EE (%)
	Alginate + CSM and CSP	CSM and CSP	L*	a*	b*	
A	3299.90 ± 54.26a	10.29 ± 3.22b	68.25 ± 2.50c	0.88 ± 0.35a	2.00 ± 0.81a	82.55
B	3325.30 ± 107.81a	11.07 ± 2.55b	69.00 ± 4.69c	1.00 ± 0.0a	1.50 ± 0.57a	84.10
C	3349.30 ± 116.48a	11.47 ± 2.92ab	70.20 ± 4.11c	1.00 ± 0.0a	1.50 ± 0.57a	85.45
D	3338.00 ± 147.67a	12.17 ± 3.17ab	74.00 ± 3.36abc	1.00 ± 0.0a	1.50 ± 0.57a	84.18
E	3292.40 ± 45.70a	11.94 ± 3.77ab	72.50 ± 6.60bc	1.00 ± 0.0a	1.75 ± 0.95a	90.26
F	3312.70 ± 107.81a	14.45 ± 3.69a	79.00 ± 2.16a	1.25 ± 0.46a	2.00 ± 0.81a	94.15
G	3342.30 ± 102.61a	13.35 ± 2.92ab	77.25 ± 2.62ab	1.13 ± 0.35a	2.25 ± 0.5a	92.24
H	3307.80 ± 138.85a	13.14 ± 3.12ab	76.75 ± 4.03ab	1.14 ± 0.38a	1.75 ± 0.92a	91.40

*Note:* Data (mean ± standard error) are from three replications (*n* = 3). Means in the same column with different lowercase letters (a–c) among the samples differ significantly (*p* ≤ 0.05). Samples: A (0,2); B (2,0); C (2,2); D (0,4); E (4,0); F (4,4); G (2,4); H (4,2) percent of CSM and CSP respectively that used in microencapsulation as wall materials.

Abbreviations: CSM, camelina seed mucilage; CSP, camelina seed protein; EE, encapsulation efficiency; MLR, microencapsulated *Lacticaseibacillus rhamnosus*.

**FIGURE 1:**
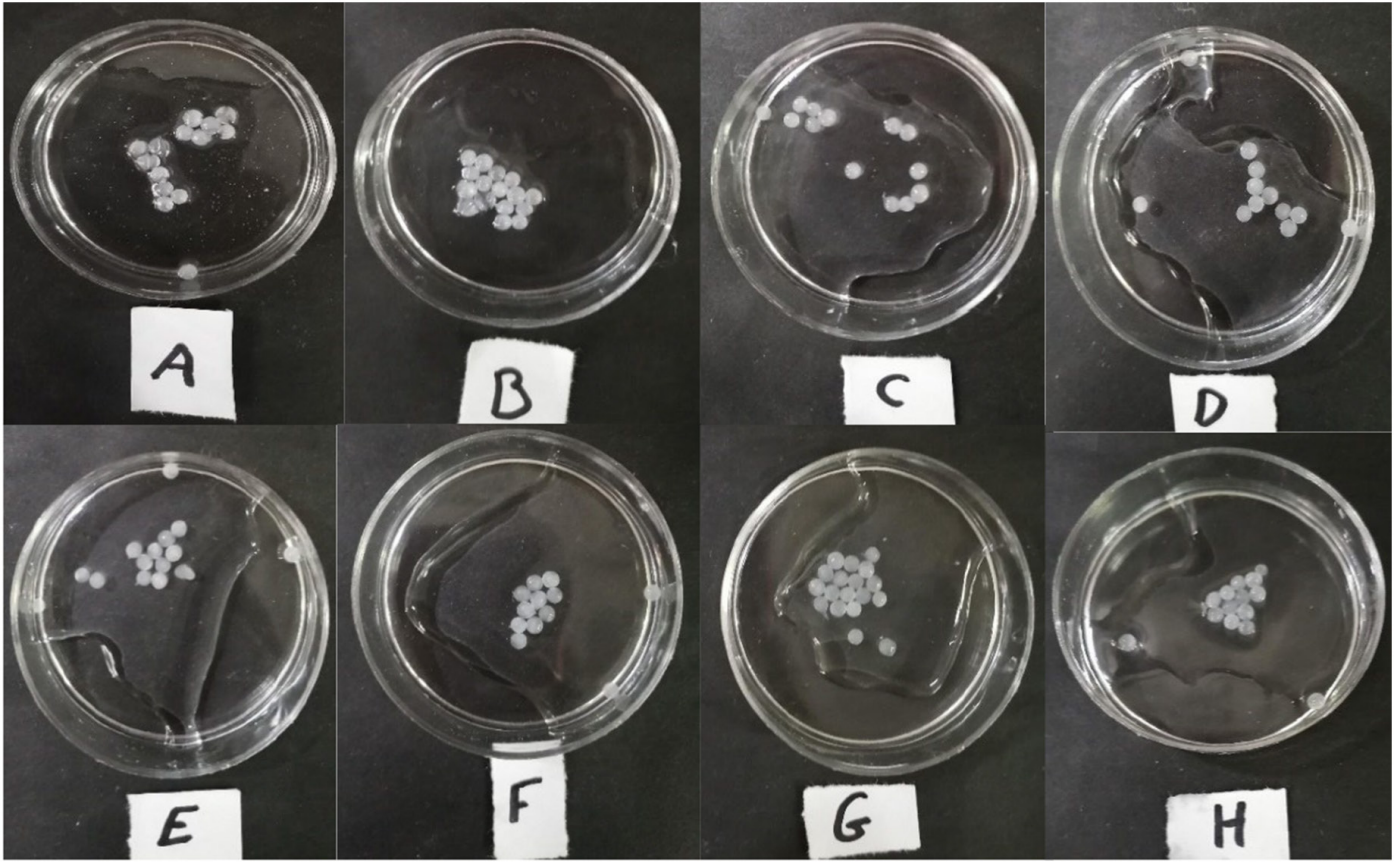
Photography images from MLR, A (0,2); B (2,0); C (2,2); D (0,4); E (4,0); F (4,4); G (2,4); H (4,2) percent of CSM and CSP respectively that used in microencapsulation as second layer of wall materials. CSM, camelina seed mucilage; CSP, camelina seed protein; MLR, microencapsulated *Lacticaseibacillus rhamnosus*.

The encapsulation efficiency (EE) of the hydrogel beads was the highest (94.15%) among the bead samples (Table 1). A previous study reported a high EE% value (94.15%) when the alginate‐gelatin hydrogel was used for probiotic encapsulation. In this work, the EE percentage was slightly lower compared to other research (Pourakbar et al. 2023). The bead morphologies are shown in Figures 1 and S1. All the beads are white and semi‐transparent except C, E, F, G, and the H beads became opaque because the wall materials contained protein (CSP) and carbohydrate (CSM).

### 3.2 Color of the Beads

One of the critical quality characteristics of the beads is the color parameter. Notably, the structure of the second layer of the beads could influence the color parameter. In this research, it was observed that as the concentrations of CSM and CSP in the beads increased, the a* and b* parameters remained constant (Table 1). The color parameters (a* and b*) remaining stable among MLR could be attributed to the colorlessness of CSM and CSP. Neither of them contributes to the color index. The degree of cross‐linking reaction between CSP, CSM, and alginate may affect the L* parameters. The higher concentration of wall materials used in the second layer of beads results in an increase in the brightness of the bead (Lopez et al. 2023).

### 3.3 Heat Resistance of MLR and FLR

**FIGURE 2:**
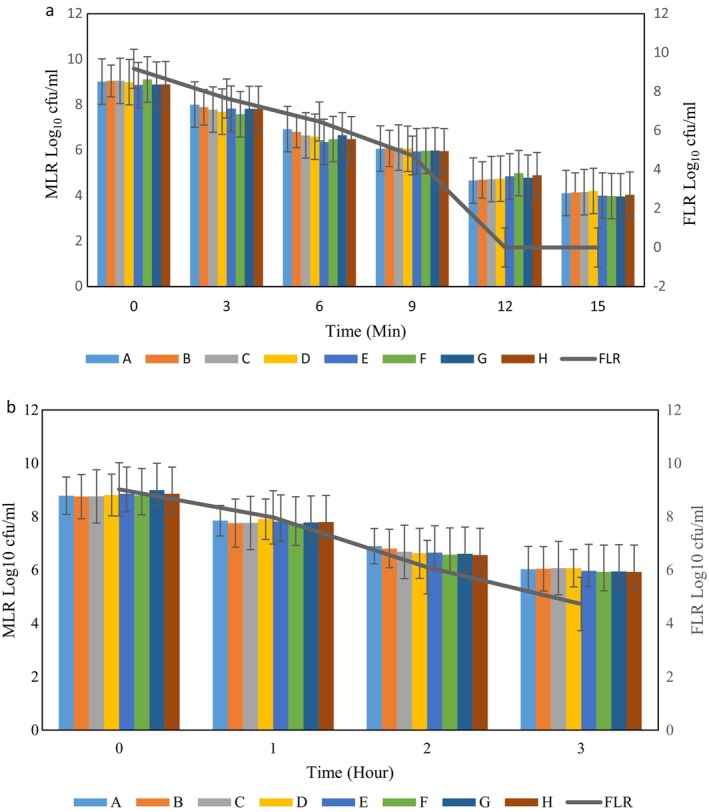
The free *Lacticaseibacillus rhamnosus* (FLR) and *microencapsulated Lacticaseibacillus rhamnosus* (MLR) survive at 72°C (a) and salt 15% w/v and pH 1.5 (b). Data (mean ± standard error) are from three replications. MLR samples consist of: A (0,2); B (2,0); C (2,2); D (0,4); E (4,0); F (4,4); G (2,4); H (4,2) percent of CSM and CSP respectively that used in microencapsulation as second layer of wall materials; CSM, camelina seed mucilage; CSP, camelina seed protein.

Microcapsules are often subjected to harsh conditions like high temperatures in processing and packaging (hot filling packaging). The survival ability of the FLR and MLR during heat stress are shown in Figure 2a. The FLR could not survive at a minimal probiotic concentration (10^6^ CFU/mL) after 2 min at 72°C. In comparison, the MLR exhibited greater viability for 3 min, while maintaining an average count of 6 Log CFU/mL. These results align with a study by Sekhavatizadeh et al. (2023), who observed that microencapsulated *Lactobacillus plantarum* with double layers consisting of alginate–galbanum (*Ferula Gummosa* Boiss) gum presented a high survival ability to heat stress (72°C) (Sekhavatizadeh, Afrasiabi, and Montaseri 2023). Moreover, one of the effective factors for the survival of *Lactobacilli* is the type and combination of wall material (He et al. 2021). Heat resistance is also influenced by the number of bead layers. The viable count of MLR and FLR decreased over time, with the decreasing rate of MLR being lower than that of FLR. There was no significant difference among FLR with different CSM and CSP concentrations. Other studies have demonstrated that employed bilayer encapsulation to protect *Lactobacillus casei* increased resistance to heat stress (Beldarrain‐Iznaga et al. 2020). Moreover, CSM can improve thermal stability (Mannai et al. 2023).

### 3.4 Acid and Salt Tolerance of FLR and MLR

As the results clearly show that the viable number of MLR was higher than FLR at salt and acid stress (Figure 2b). Moreover, the survival rate of MLR (F sample) at 67.28% was higher than that of FLR at 59.22%. These findings suggested that these probiotics survive in osmolarity and acid conditions. Using alginate‐CSM‐CSP as wall materials provides more LR resistance to acid and salt stress. An increase in osmolarity and reduction in water activity may be attributed to LR survival during storage. Salts are commonly incorporated into food to enhance the taste or to prevent spoilage. However, there is limited knowledge regarding how microencapsulated probiotics withstand high salt concentrations (Amira et al. 2019).

### 3.5 SEM of Beads

**FIGURE 3:**
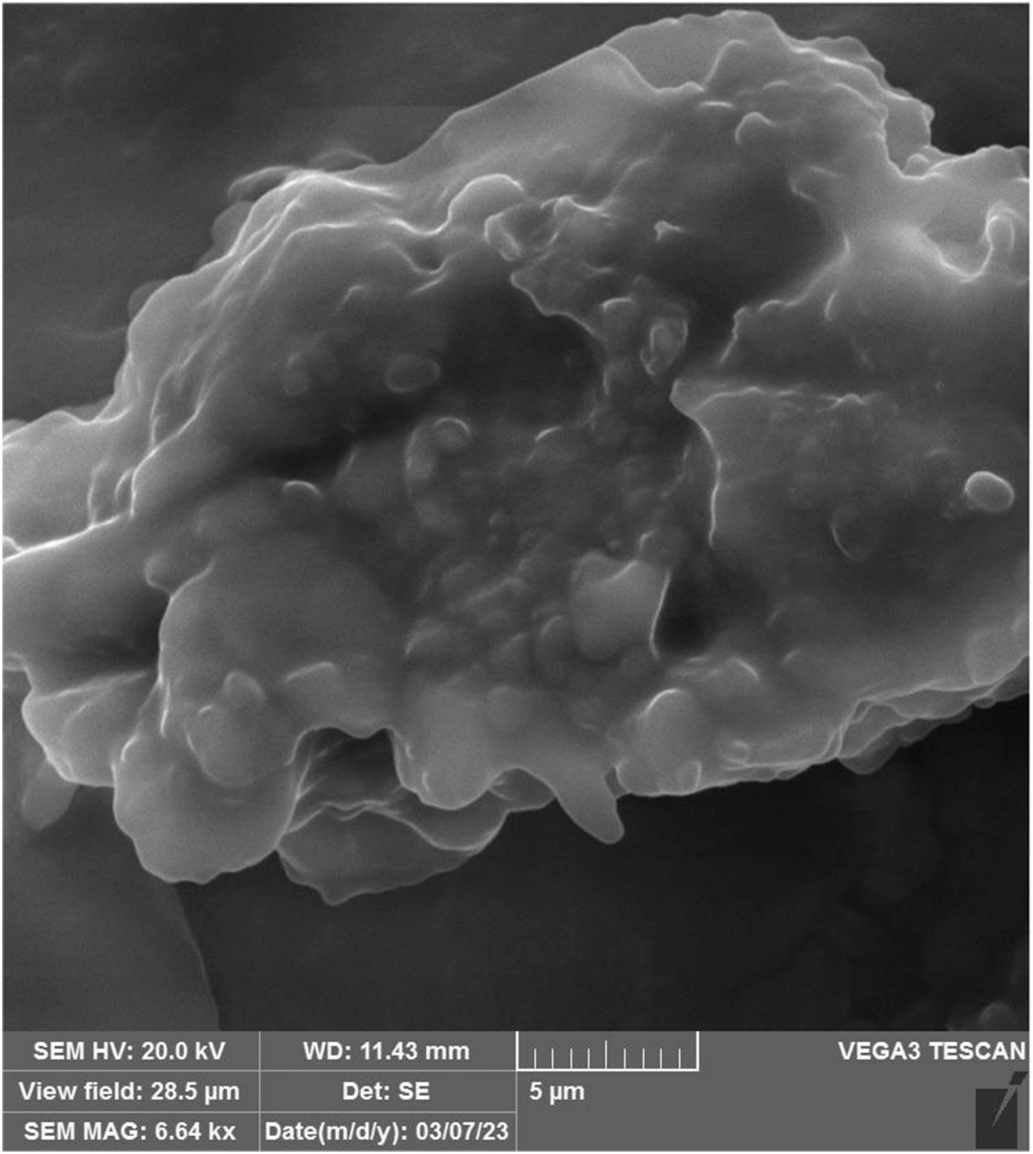
SEM image of MLR (F sample) contains (4,4) percent of CSM and CSP respectively that used in microencapsulation. CSM, camelina seed mucilage; CSP, camelina seed protein; MLR, microencapsulated *Lacticaseibacillus rhamnosus*.

The morphology of CSM‐CSP (4,4) % w/w LR beads is presented in Figure 3. However, the bead had a rounded oval shape, which may be attributed to the wall materials diversity. Furthermore, this may be related to the interactions between alginate and CSM‐CSP. No pores and cracks were seen on the surfaces of the beads, which demonstrated bead structure integrity. Therefore, it may enhance the encapsulation efficiency and probiotic survival ability. The bead surface's integrity could improve probiotic viability during the storage time and GI condition (Zeng et al. 2023; Zhang et al. 2023). Azeem et al. (2023) reported that solid lipid microparticles encapsulated by whey protein and Gum Arabic containing probiotic bacteria present a smooth surface, a spherical shape, and crystallized material with variable diameters in SEM image (Azeem et al. 2023). The differences in the results of this study compared to our own may be attributed to variations in the coating techniques and the types of materials employed in the wall construction.

### 3.6 Titratable Acidity and pH of SMD

**FIGURE 4:**
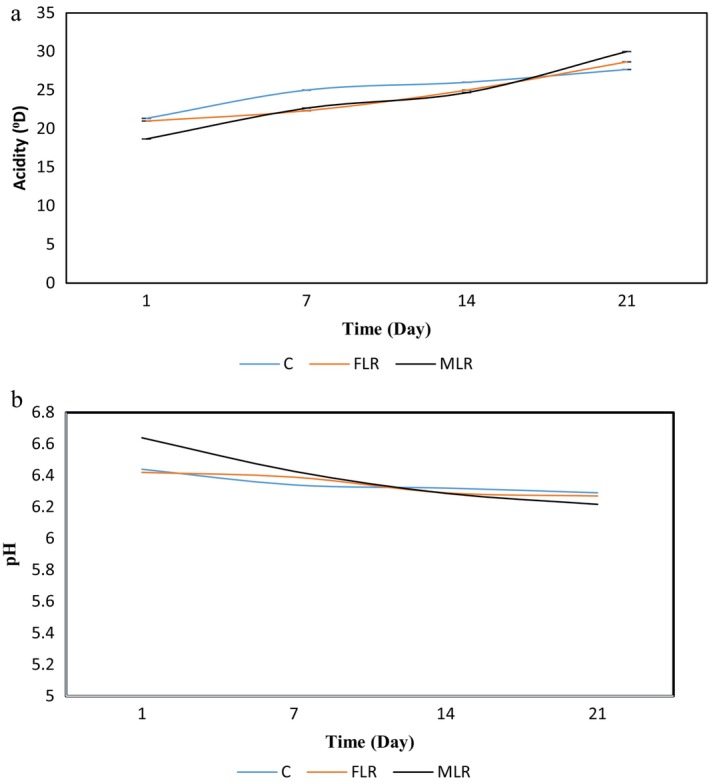
The pH (a) and acidity (b) of free *Lacticaseibacillus rhamnosus* (FLR) and microencapsulated *Lacticaseibacillus rhamnosus* (MLR) survive during storage time.

The MLR and FLR acidity was higher than the C sample. Additionally, LR influences the pH of the SMD (Figure 4a). LR is a homofermentative probiotic strain that produces lactic acid from lactose, which explains this higher acidification and lower pH in the supplemented SMD. However, no adverse effect was observed in the product (D'ambrosio et al. 2023).

The findings indicate that during fermentation, higher lactose conversion in probiotic foods leads to increased production of organic acids. Microorganisms break down carbon sources to release energy in the fermentation process. In microbial fermentation, alcohol, propionic, lactic, and acetic acids are the primary end products. Furthermore, the production may depend on the strain type (Rosa et al. 2023).

According to the findings, the acidity was lower, and the pH was higher in MLR and FLR, respectively, because of the decreased activity of MLR (Figure 4a,b). The metabolism of the microcapsules in the food was affected by the size of the alginate layer. The more the layers in the bead, the less the acid that was released. In this research, a two‐layer bead was used. Furthermore, the multiple outer layers may slow food intake, leading to a reduction in the released organic acid (Ghasemnezhad et al. 2017).

Other researchers presented comparable results. For instance, the new probiotic whey beverage was produced by microencapsulated *Lactobacillus plantarum* in alginate, inulin, and dextran. Lower acidity production was seen in microencapsulated form (Saniani, Nateghi, and Hshemiravan 2023).

### 3.7 Survival of FLR and MLR in GI

**FIGURE 5:**
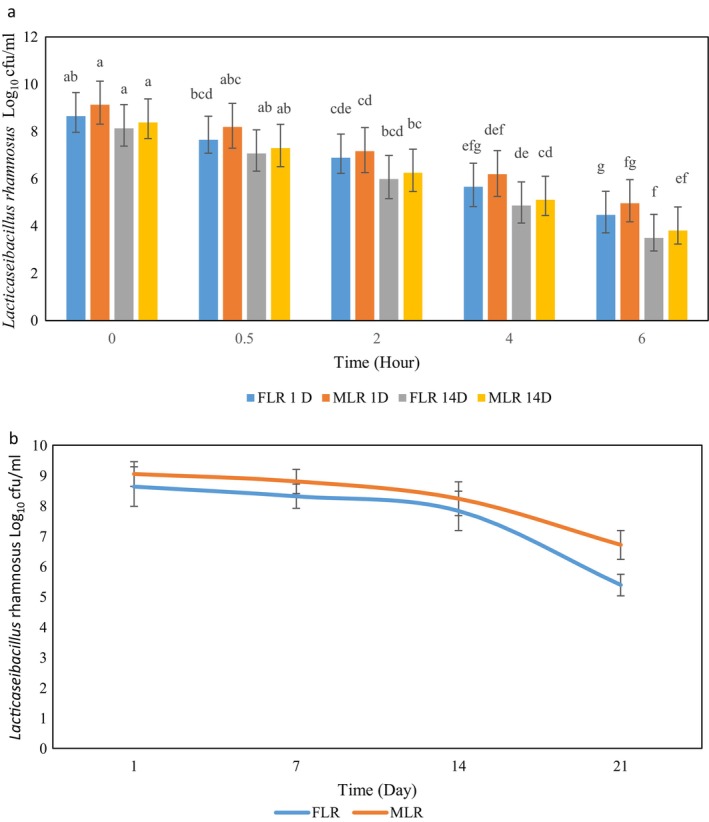
The free *Lacticaseibacillus rhamnosus* (FLR) and microencapsulated *Lacticaseibacillus rhamnosus* (MLR) survive during simulation gasterointestinal condition (a); storage time (b) in saffron milk dessert. Data (mean ± standard error) are from three replications.

The number of viable FLR and MLR was reduced in simulated gastric fluid (SGF; Figure 5a). After undergoing SGF and simulated intestinal fluid (SIF) treatment for 360 min, the viability of FLR and MLR decreased from 8.65 to 4.47 log (CFU/g) and from 9.13 to 4.96 on the first day of storage, respectively. It has been observed that LR exhibited sensitivity toward the acidic content of the gut. As reported in many research studies, gastric acid can quickly destroy free probiotics. Under the same conditions, the decrease rate in free bacterial cells was more significant than the single or double‐layer probiotic beads. Microencapsulation is a highly effective method for protecting probiotics from acidic, harsh conditions. It may enable more probiotics to reach the intestine and exert beneficial effects. In a similar research, LR showed rapid release from beads at 30 min in SIF. The viable cell number was 9.10 log (CFU/g) after treatment at 90 min in SIF (Chen et al. 2023). In prior research, the viable cell counts of *Limosilactobacillus reuteri* within spray‐dried microcapsules composed of alginate/whey protein isolate fibrils/shellac/sucrose were measured at 2.38 ± 0.38 log CFU/mL in SGF and 1.84 ± 0.13 log CFU/mL in SIF. The protective effect of S/O/W microcapsule in SGF and SIF was more valuable than the results of the present investigation. The improved probiotic survival in S/O/W/S/S microcapsule in digestive juices might be attributed to the physical barriers of butterfat and whey protein isolate fibrils (Guo et al. 2024).

The findings of this research showed that microencapsulation confers an increased level of survival against SGF conditions. The formation of the bead and bacterial entrapment were significant factors in protecting probiotics against the gastric enzymes and their low pH (Sekhavatizadeh, Afrasiabi, and Montaseri 2023). Moreover, CSM and CSP may prevent the diffusion of bile salt solution into the beads. The survival of MLR and FLR significantly decreased (*p* ≤ 0.05) with GI fluid treatment for the first 360 min. Various combinations of wall materials can enhance the survival of probiotics during gastrointestinal transit. For instance, *E. mundtii* SRBG1 was better protected through microencapsulation using a combination of inulin and maltodextrin as wall materials when exposed to GI (Sakoui et al. 2022).

### 3.8 Storage Stability of SMD

The viable numbers of LR during 21 days of storage are displayed in Figure 5b. The data showed that LR decreased in all samples at 4°C during storage. The viable numbers were 6.7 and 5.38 log CFU/g for MLR and FLR, respectively, which displayed a decrease of 2.34 and 5.38 log CFU/g after 21 days of storage. The results indicate that using alginate‐CSM‐CSP in the coencapsulation of LR affects the survival rate of MLR. It is crucial to emphasize that the type of wall material used as the second layer can affect the probiotic survival ability during storage (Kouamé et al. 2023). Many proteins are commonly utilized as matrix martial because they possess properties that serve as an important deterrent against oxygen and carbon dioxide permeability (Safeer Abbas et al. 2023). Moreover, Camelina proteins have hydrophobic regions, which can interact with the hydrophilic polysaccharides in CSM. Additionally, since mucilage is anionic due to its polysaccharide composition, it might form electrostatic interactions with cationic or positively charged regions of proteins (Ghosh and Bandyopadhyay 2012). In a similar investigation, the survival of *Lactobacillus plantarum* decreased significantly at the end of storage time. Moreover, yogurt containing free *B. bifidum* exhibited a significant decrease in viable cell counts compared to its encapsulated form using sodium alginate or whey protein over the duration of storage (Afzaal et al. 2022). So, microencapsulated probiotic bacteria can survive better in food during storage time (Pourakbar et al. 2023).

### 3.9 Texture, Color, and SEM of SMD

The TPA of SMD samples is presented in Table 2. The hardness increased in all samples, especially MLR. The pH of MLR‐SMD samples affected the hardness parameter. The lower the pH, the higher the hardness of the MLR‐SMD samples. The casein strands become protonated due to the acidic conditions Afterward, the reaction intensifies the hydrophobic interactions between protein molecules, leading to increased curd hardness (Sekhavatizadeh, Afrasiabi, and Montaseri 2023). Therefore, the MLR sample with the lowest pH (6.2) (Figure 4a) had greater hardness in the 21^st^ storage time.

**TABLE 2:** Color and texture analysis in supplemented SMD samples during storage time.

Physical parameters	Samples	Day 1	Day 28
(L*)	C	71.5 ± 1.55a	68.25 ± 0.85a
	FLR	68.0 ± 0.91a	67.0 ± 1.41a
	MLR	70.25 ± 1.18a	74.75 ± 3.54a
(a*)	C	3.0 ± 0.40a	3.75 ± 0.85a
	FLR	4.25 ± 0.75a	3.75 ± 0.62a
	MLR	4.75 ± 0.95a	3.75 ± 0.47a
(b*)	C	65.75 ± 0.47ab	65 ± 0.70b
	FLR	63.75 ± 1.49b	66 ± 0.70b
	MLR	68.0 ± 1.08a	72.50 ± 2.50a
Hardness (g)	C	45.25 ± 1.25a	112.38 ± 3.12b
	FLR	52.75 ± 0.75a	138.10 ± 8.60c
	MLR	52.60 ± 2.10a	313.70 ± 0.80d
Adhesiveness (mJ)	C	0.54 ± 0.02a	1.80 ± 0.07a
	FLR	0.90 ± 0.11a	1.57 ± 0.13a
	MLR	1.07 ± 0.02a	2.01 ± 0.98a
Cohesiveness	C	0.57 ± 0.07ab	0.78 ± 0.02a
	FLR	0.36 ± 0.1b	0.52 ± 0.16ab
	MLR	0.65 ± 0.01ab	0.47 ± 0.08ab
Chewiness	C	1.23 ± 0.19c	4.85 ± 0.02b
	FLR	0.87 ± 0.19c	4.39 ± 0.19a
	MLR	1.07 ± 0.01c	9.36 ± 0.46b
Springiness (mm)	C	4.87 ± 0.33b	9.30 ± 0.18a
	FLR	4.71 ± 0.36b	8.70 ± 0.42a
	MLR	5.06 ± 0.03b	8.22 ± 0.88a
Gumminess (g)	C	25.75 ± 2.355 cd	52.40 ± 2ab
	FLR	19.20 ± 5.50d	47.80 ± 1.10b
	MLR	32.55 ± 0.14c	58.80 ± 1.40a

*Note:* Data (mean ± standard error) are from three replications (*n* = 3). Lowercase letters (a–d) show significant differences (*p* ≤ 0.05) among samples in each parameter during storage time.

Abbreviations: C, control were the saffron milk dessert (SMD) samples; FLR, free *Lacticaseibacillus rhamnosus*; MLR, microencapsulated *Lacticaseibacillus rhamnosus*.

Differentiating a semi‐solid food up to becoming ready for swallowing is called gumminess. Hardness and cohesiveness parameters affect gumminess (Sekhavatizadeh, Afrasiabi, and Montaseri 2023). Since these factors have increased during storage time, the gumminess value for all types of SMD was increased.

The results also showed that in the supplemented SMD and control, cohesiveness, and adhesiveness values were constant, but springiness increased during the storage time.

The secondary textural parameter of food is chewiness. It is “the number of chews required to be ready to swallow the sample”. Proper chewing ability guarantees the pleasure of food tasting and boosts mouthfeel. In a study, the cheese springiness increased but chewiness, cohesiveness, and adhesiveness values remained constant during the storage time (Sekhavatizadeh et al. 2023).

The primary parameters that have a significant impact on chewiness are gumminess and springiness. An increase in gumminess and springiness content increased chewiness in the cheese (Borhanpour et al. 2021). Therefore, the MLR chewiness value was higher than FLR but equal to the control sample.

The L*, a*, and b* are the color index of food that presents whiteness, redness, and yellowness, respectively. The insertion of MLR and FLR did not significantly decrease color parameters during the storage time (*p* > 0.05; Table 2). Therefore, among the samples, FLR and MLR whiteness were equal. This is probably because these desserts were not able to absorb water. After all, the L* value is correlated with the water existing on the food surface. The LR content is too low to modify whiteness in FLR‐ SMD. A significant difference in the b* parameter was seen in MLR‐SMD at the 21st day of storage (*p* ≤ 0.05). In low pH, an increase in a* and b* values and a decrease in L* value were seen. These changes were attributed to the casein micelles reduction size. The casein micelles are achieved by scattering light in the visible spectrum. Therefore, the pH values and color parameters are correlated (Karimi, Sekhavatizadeh, and Hosseinzadeh 2021).

**FIGURE 6:**
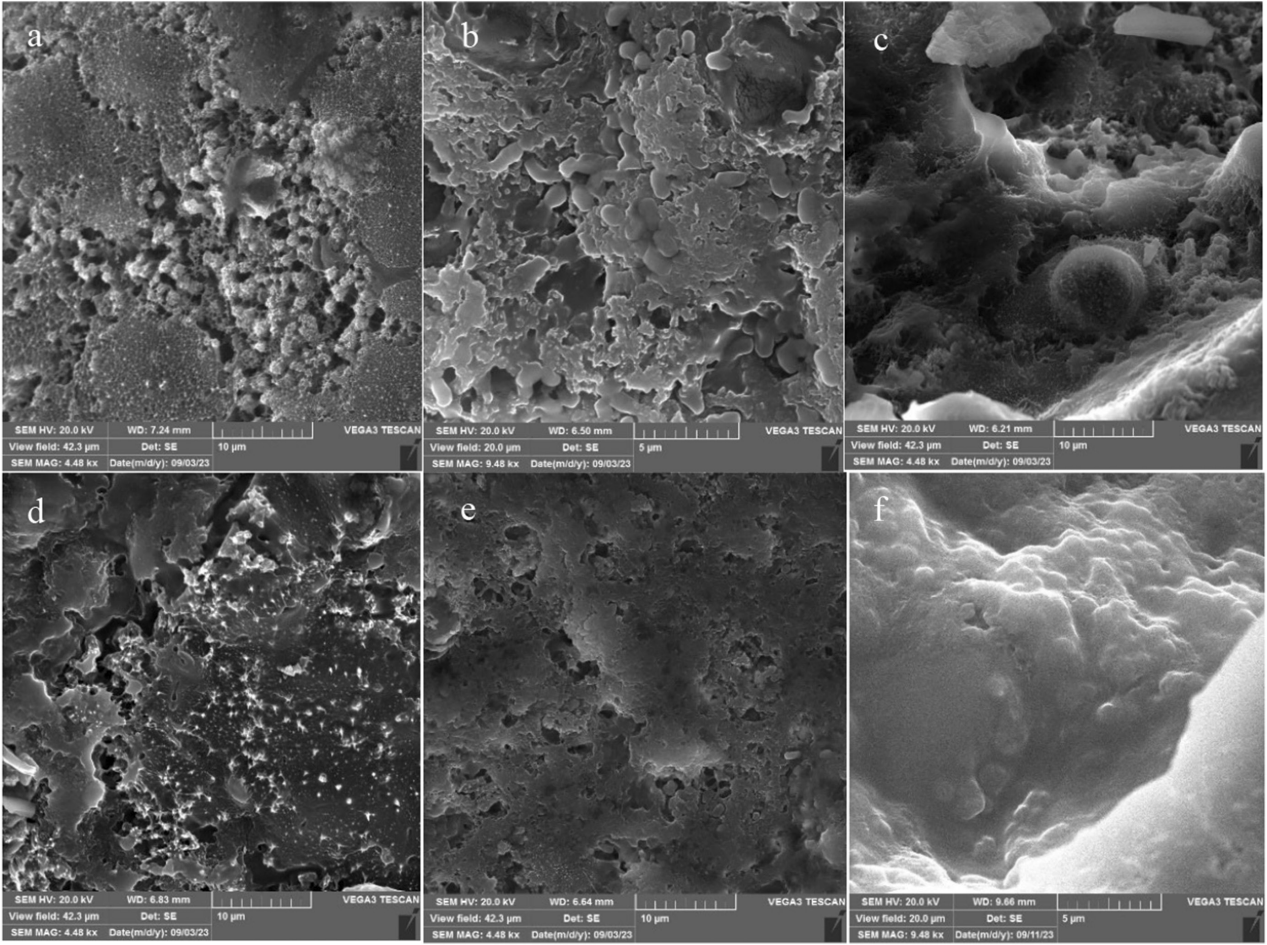
The SEM image of saffron milk dessert contain the free *Lacticaseibacillus rhamnosus* (FLR) and *microencapsulated Lacticaseibacillus rhamnosus* (MLR) and Control (C), during storage time. C in 1st (a); FLR in 1st (b); MLR in 1st (c); C in 28th (d); FLR in 28th (e); MLR in 28th (f) of storage time.

The SEM images were applied to investigate microstructural changes among C, MLR, and FLR‐SMD samples during the storage. The images were obtained from the C, MLR, and FLR‐SMD samples on the first (Figure 6a–c) and last day of the storage time (Figure 6d–f). SEM images revealed significant changes in the structure and morphology of FLR, MLR, and C‐SMD on the first day of storage. On day 1, the C‐SMD sample had a protein network with more cavities, and a less dense structure (Figure 6a). However, the MLR and FLR‐SMD samples had a denser structure and fewer cavities. Similarly, studies have shown that a gelatinous formation was detected in the yogurt in the first days of storage, a hypothesized result from the protein networks (Pourakbar et al. 2023). The globular protein aggregate structure was seen in MLR‐SMD, presumably due to CSM and CSP acting as protein binders to SMD protein. These materials absorb water and expand, filling the cavities. It will continue to swell during storage. Some solubilizing molecules within the CSP and CSM may enter the SMD protein. So, the open structure of the protein micelle network was revealed (Sandoval‐Castilla et al. 2004).

## 4 Conclusion

This study focused on the encapsulation of LR using eight different combinations of varying concentrations of encapsulants (CSM and CSP). The encapsulation efficiency was found to be dependent on the encapsulants matrix employed. SEM analysis confirmed that the encapsulation was successful. The microencapsulation of LR using optimal concentrations of CSP and CSM significantly maintained the quality attributes of SMD and maintained the viability of LR during storage. Encapsulation is recognized as an effective method for delivering probiotic bacteria to the GI. This demonstrated the effectiveness of combining CSM and CSP to encapsulate substances in harsh conditions. The results also revealed that CSM and CSP formed composite gel network structures that exhibited improved texture and stability within the SMD. Consequently, it is imperative to encapsulate LR cells prior to their incorporation into dairy products.

## Author Contributions

**Mohammad Ganje:** project administration (equal). **Seyed Saeed Sekhavatizadeh:** writing – original draft (equal). **Fatemeh Teymouri:** visualization (equal). **Mostafa Gilkheiri:** software (equal). **Bentalhoda Rahmani:** writing – review and editing (equal).

## Ethics Statement

The authors have nothing to report.

## Conflicts of Interest

The authors declare no conflicts of interest.

## Supporting information


Figure S1.
Table S1.

## Data Availability

Data will be made available on reasonable request.
