# Supplementary material for: Encapsulation of Lacticaseibacillus rhamnosus by Extrusion Method to Access the Viability in Saffron Milk Dessert and Under Simulated Gastrointestinal Conditions
Source: Food Sci Nutr. 2024 Oct 23;12(11):9714–26. doi: 10.1002/fsn3.4510 (PMC11606897; doi:10.1002/fsn3.4510)
Supplement: Supplementary file 1 — Figure S1. Table S1. [file FSN3-12-9714-s001.docx]

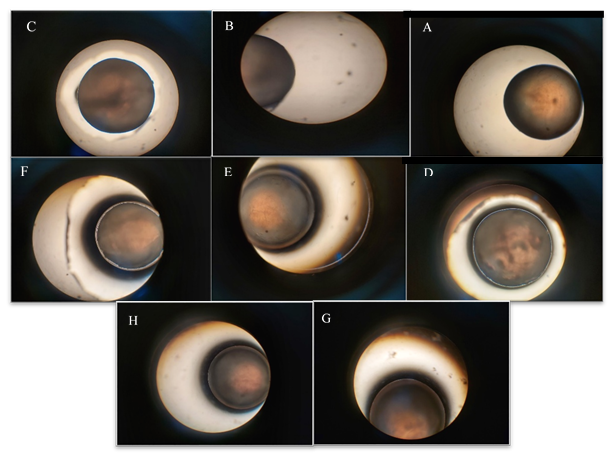


Fig 1S. Light microscopy images of MLR, A (0,2); B (2,0); C (2,2); D (0,4); E (4,0); F (4,4); G (2,4); H (4,2) percent of CSM and CSP respectively that used in microencapsulation(40X).

Microencapsulated *Lacticaseibacillus rhamnosus* (MLR); camelina seed mucilage (CSM); camelina seed protein (CSP)

Table 1s: The second layer component of MLP by extrusion

| CSP (%) | CSM (%) | Treatment |
| --- | --- | --- |
| 2 | 0 | A |
| 0 | 2 | B |
| 2 | 2 | C |
| 4 | 0 | D |
| 0 | 4 | E |
| 4 | 4 | F |
| 4 | 2 | G |
| 2 | 4 | H |

Camelina seed mucilage (CSM); Camelina seed protein (CSP)
